# Differential shooting training in youth basketball players: an analysis of performance effects

**DOI:** 10.3389/fpsyg.2025.1709103

**Published:** 2025-11-20

**Authors:** Grėta Burkaitė, Bruno Figueira, Wolfgang Schöllhorn, Diogo Coutinho, Rūtenis Paulauskas

**Affiliations:** 1 Educational Research Institute, Education Academy, Vytautas Magnus University, Kaunas, Lithuania; 2 Departamento de Desporto e Saúde, Escola de Saúde e Desenvolvimento Humano, Universidade de Évora, Évora, Portugal; 3 Comprehensive Health Research Centre (CHRC), Universidade de Évora, Évora, Portugal; 4 Department of Training and Movement Science, Institute of Sport Science, Johannes Gutenberg-University Mainz, Mainz, Germany; 5 Research Center in Sports Sciences, Health Sciences and Human Development, CIDESD, University of Maia, UMAIA, Maia, Portugal

**Keywords:** movement variability, shot accuracy, youth athlete development, small-sided games, perceived exertion

## Abstract

**Introduction:**

Differential Learning introduces increased variability during practice to enhance motor skill acquisition.

**Methods:**

This study investigated the effects of Differential Training (DT) (Differential Training Group (DTG): *n* = 19, age = 13.1 ± 0.19 years, height = 170.1 ± 9.5 cm, body mass = 56.9 ± 9.7 kg, training experience = 6.4 ± 1.3 years, maturity offset = 0.7 ± 0.8 years) on shooting accuracy and 1 × 1 small-sided game performance, compared to Traditional Training (TT) [Traditional Training Group (TTG): *n* = 18, age = 13.8 ± 1.1 years, height = 171.3 ± 8.6 cm, body mass = 59.4 ± 15.4 kg, training experience = 6.5 ± 1.5 years, maturity offset = 0.5 ± 1.1 years], in youth basketball players for an 8-week intervention (16 sessions). Outcomes included 2-point (2-pts) and 3-point (3-pts) shooting accuracy test (BJSAT), 1 × 1 scoring performance, stationary shooting accuracy test (SSAT), and rate of perceived exertion (RPE).

**Results:**

Linear mixed-model analyses revealed that DT improved two-point BJSAT relative to TT at post-test (*β* = −2.48; *p* = 0.042) and gains were maintained at retention (*p* = 0.001). Three-point BJSAT improved over time in both groups (*p* = 0.004) with no between-group difference at retention. 1 × 1 SSG scoring increased over time (*p* < 0.001) with no between-group effect at retention. DT outperformed TT in the 30-shot task (Δ = 3.11, 95% CI [1.59, 4.63]; *p* < 0.001) and elicited lower RPE (Δ = −0.96, 95% CI [−1.47, −0.46]; *p* < 0.001).

**Discussion:**

These results indicate a superior efficacy of DT for improving shooting performance and managing perceived effort. The differential adaptation rates between mid- and long-range shooting highlight the value of movement variability in skill learning. However, limited transfer to SSG outcomes suggests further research is needed to optimize DT protocols for complex game contexts.

## 1 Introduction

Shooting is a cornerstone skill in basketball, influencing both individual and team outcomes (Erčulj and Štrumbelj, 2015). Even minor gains in shooting accuracy can significantly affect performance, particularly in youth-level games (Ortega et al., 2006). However, basketball’s dynamic nature presents players with constant challenges, including changes in positioning, timing, and available shooting space (Courel-Ibáñez et al., 2017). To address these complexities, coaches must incorporate sensitive strategies into training design, adapting tasks to reflect real-game variability. To improve skill adaptability under these variable conditions, training is recommended to also encourage creativity and perceptual responsiveness (Santos et al., 2018). This study draws on complementary perspectives in motor learning. Specifically, variable practice within schema theory (Schmidt, 1975) and the contextual interference effect (Shea and Morgan, 1979) concern how variation and practice scheduling can influence retention/transfer, while structural learning addresses how learners extract invariant structure across related tasks (Braun et al., 2010). We note that most evidence for these principles comes from simplified laboratory tasks; therefore, they do not by themselves prescribe ‘ecologically valid’ practice. Our rationale for employing varied, game-relevant tasks is instead grounded in dynamical systems / ecological-dynamics accounts, in which adaptable behavior emerges from the interaction of organism, task, and environment constraints (Warren, 2006; Frank et al., 2008; Birrento Aguiar et al., 2023).

While these principles and perspectives can be leveraged to promote learning and adaptability, they have typically been tested under predictable conditions, which may limit their direct generalization to complex game environments. For example, it is expected that by increasing the number of available targets, players would enhance the number of shooting opportunities, while also decreasing the distance to the nearest opponent. In this respect, evidence from the last decade has been suggesting of variability as a mean to promote technical development, creativity and to encourage adaptive movement responses resulting from the different configurations of play (de Souza et al., 2025; Mikalonytė et al., 2022). Under this scope, Schöllhorn (1999) introduced the Differential Learning (DL) approach, an evidence-informed framework designed to amplify variability and stochastic perturbations within training environments. The optimal magnitude of this variability is contingent upon individual characteristics and situational demands. Central to DL is the deliberate introduction of movement fluctuations during skill acquisition, achieved without reliance on repetition or prescriptive correction (Schöllhorn et al., 2012). As a fundamentally nonlinear approach, DL requires learners to execute full, context-rich motor patterns while continuously adapting to unpredictable, internally and externally imposed disturbances (Schöllhorn et al., 2012). Grounded in dynamical systems theory, DL represents a paradigm shift from prescriptive, technique-centered models toward emergent performance patterns shaped by interacting internal and external boundary conditions (Frank et al., 2008). To operationalize these principles, DL integrates metric and topological variations across practice tasks. This creates a dynamic training environment characterized by unstable inputs and outputs—commonly described as “noisy” (Santos et al., 2018; Schöllhorn et al., 2012). Such instability fosters exploratory behavior and can initiate self-organizing processes, enabling learners to discover individualized, task-specific movement solutions without having been told elements of the solution or having been guided there by restrictive exercises (Schöllhorn et al., 2006). From a dynamical systems perspective, movement variability is not only inevitable but also functionally essential. It facilitates adaptive responses to environmental changes and sustains the system complexity required for resilient performance (Frank et al., 2008). In applied contexts, manipulations such as modifying body position, ball type, or environmental conditions can enhance an athlete’s adaptive capacity during gameplay (Gaspar et al., 2019). These principles align with broader frameworks of motor system variability, effectively bridging theoretical constructs with applied training design (Schöllhorn, 2000).

DL has been effectively implemented across a range of domains, including team sports (Mateus et al., 2015; Coutinho et al., 2018), individual disciplines (Schollhorn et al., 2009; Schöllhorn et al., 2007), recreational activities (Pabel et al., 2018; James and Conatser, 2014), and medical rehabilitation (Gokeler et al., 2019; Repšaitė et al., 2015). Compared to traditional training methods, DL has demonstrated enhanced acute performance outcomes, including improvements in countermovement jump height, explosive power, linear speed, football kicking velocity, and scoring accuracy in high-pressure zones (Gaspar et al., 2019). In addition to these physical performance benefits, DL influences neuromotor and cognitive functioning. Experimental studies involving rope skipping have shown that DL elicits elevated cognitive workload and sympathetic nervous system activation, indicating greater mental demands than repetitive practice protocols (John et al., 2022). Emerging evidence also supports DL’s role in promoting long-term skill retention. For instance, in a controlled study of futsal goal-kicking, athletes who trained under DL conditions with an external attentional focus significantly outperformed those using conventional methods in retention assessments (Oftadeh et al., 2021). DL further enhances skill transfer to novel or unanticipated contexts. This is largely attributed to its foundational emphasis on adaptability. Such adaptive capacity is essential for sustained performance in dynamic environments, where task boundary conditions and situational demands are constantly evolving (Schöllhorn et al., 2006).

Basketball is distinct from other sports due to its high scoring frequency, the requirement of ball dribbling, and its emphasis on verticality, particularly jumping ability. Research examining the application of DL in basketball remains limited. To date, only a limited number of studies have evaluated its impact on basketball-specific technique training (Poureghbali et al., 2020; Schönherr and Schöllhorn, 2003). In a pre-post-test design, a repetition-based group of youth basketball players was compared with a differentially training group of similar age (Poureghbali et al., 2020). Both groups trained once a week for 30 min basketball free throw according to the group conditions. The posttest showed a highly significant higher performance improvement than the classically training group. In another investigation (Schönherr and Schöllhorn, 2003), players participated in small-sided games (SSGs) incorporating varied numerical player configurations during tasks. This intervention was associated with increased dribbling frequency and a reduced spatial exploration index, outcomes interpreted as indicative of enhanced decision-making under dynamic, game-like conditions (Schönherr and Schöllhorn, 2003). Although the initial results are promising, the study presents several methodological limitations. The sample size was small (*n* = 8), the intervention lasted only 4 weeks, and the protocol did not address precision-dependent skills, such as basketball shooting. While current evidence suggests potential benefits of DL in basketball contexts, further methodologically rigorous studies are needed. Future research should extend intervention durations and incorporate a broader spectrum of task constraints to more comprehensively assess DL’s efficacy, particularly in improving shooting performance.

The integration of DL into variation-based skill training represents a meaningful innovation in basketball shooting methodology. By leveraging contextual variability, coaches can tailor training conditions more precisely to meet each athlete’s functional performance needs. However, despite increasing theoretical support, empirical evidence evaluating DL in the context of basketball shooting remains sparse. Thus, this study aims to assess the efficacy of DT in improving shooting performance. Specifically, it investigates whether DT leads to greater improvements in mid-range and long-range jump shots, as well as shooting accuracy during one-on-one (1 × 1) game situations, compared to conventional training protocols. It is hypothesized that DT will lead to significantly greater improvements in spot-up shooting accuracy compared to TT, while also resulting in lower levels of perceived exertion during training.

## 2 Materials and methods

### 2.1 Subjects

*A priori* power analysis was conducted using G*Power software (Version 3.1.9.6; Institut für Experimentelle Psychologie, Düsseldorf, Germany) to determine the required sample size. Based on an expected effect size of 0.7, an alpha level (*α*) of 0.05, and a statistical power (1 − *β*) of 0.80, the minimum required sample was estimated at 18 participants per group. Participants were randomized at the individual level to the Differential Training Group (DTG) or the Traditional/Repeated Training Group (TTG) in a 1:1 ratio using a computer-generated list (simple randomization; no stratification/blocking). The randomization sequence was generated by a study collaborator not involved in recruitment, baseline testing, intervention delivery, or outcome assessment. Allocation was concealed using sequentially numbered, opaque, sealed envelopes (SNOSE) prepared off-site from the testing venue. Envelopes were tamper-evident, identical in appearance, and opened in numerical order after written consent and completion of all baseline assessments, immediately before the first training session. Participant enrollment was conducted by the site investigator; envelope opening and assignment logging were performed by an administrative staff member who did not participate in testing or coaching; no crossovers occurred. Eligibility criteria included at least 4 years of formal basketball training and competition experience. Following these exclusions, the final sample consisted of 37 trained basketball players. The DTG group (*n* = 19) had a mean age of 13.1 ± 0.19 years, height of 170.1 ± 9.5 cm, body mass of 56.9 ± 9.7 kg, training experience of 6.4 ± 1.3 years, and a maturity offset of 0.7 ± 0.3 years. The TTG group (*n* = 18) had a mean age of 13.8 ± 1.1 years, height of 171.3 ± 8.6 cm, body mass of 59.4 ± 15.4 kg, training experience of 6.5 ± 1.5 years, and a maturity offset of 0.5 ± 1.1 years. Maturity offset was calculated for each athlete using the predictive equation developed by Moore et al. (2015). There were no significant differences between the DTG and TTG baseline characteristics (*p* > 0.05). All participants were concurrently enrolled in the Lithuanian Basketball Federation Youth National Development Program. Throughout the study, both groups followed the same federation training schedule and content, delivered by the same staff and at the same venues: three 90-min team sessions per week, each comprising ~30 min of technical skill instruction, ~20 min of small-sided games (1v1–3v3), and ~30 min of continuous 5v5 gameplay. The experimental sessions (DTG vs. TTG) were implemented in addition to this routine and were volume-matched within the protocol (identical number of shots, court angles, and distances). Thus, aside from the randomized intervention, co-training exposure was equivalent by design across groups. Informed consent was obtained from all participants and their legal guardians. Participants were informed of their right to withdraw from the study at any time without consequence. The study protocol received ethical approval from the Institutional Ethics Committee of Vytautas Magnus University (Approval No. SA-EK-24-42), in accordance with the principles of the Declaration of Helsinki.

### 2.2 Experimental procedure design

Each group completed three testing sessions: a pre-test, a post-test, and a retention-test. The effects of DT were evaluated using two validated performance measures: the Basketball Jump Shooting Accuracy Test (BJSAT) and a one-on-one (1v1) SSG shooting assessment. To examine the acute effects of DT, participants completed the Stationary Shooting Accuracy Test (SSAT) and reported their Rate of Perceived Exertion (RPE) immediately before and after a single training session. Prior to each testing and training session, all participants performed a standardized 15-min warm-up protocol that included running, ball-handling drills, shooting exercises, and dynamic stretching. All sessions were conducted on indoor hardwood basketball courts. Standardized size 6 basketballs (Spalding Precision TF-1000) were used consistently throughout the study period.

#### 2.2.1 Training intervention

**Figure 1:**
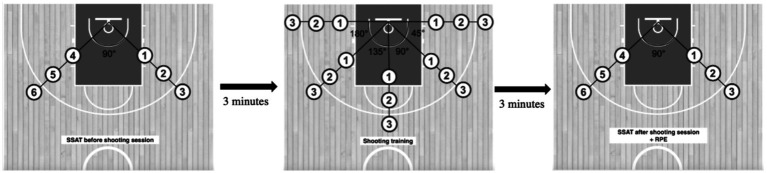
Schematic representation of the intervention and data collection procedure.

The training intervention spanned 8 weeks and was delivered during the regular basketball season. Participants completed two on-court training sessions per week as part of the experimental protocol. In each session, DT was implemented immediately following a standardized warm-up routine. Each DT session consisted of a single set of 30 shots executed from five court angles: 0°, 45°, 90°, 135°, and 180°. At each angle, participants attempted six shots, two from each of three fixed distances: 3.90 m (Position 1), 5.30 m (Position 2), and 6.75 m (Position 3). Each shot was performed under a distinct constraint designed to introduce movement variability and task-specific perturbation (Figure 1; Table 1). Both DTG and TTG sessions were preceded by the SSAT, administered 3 min before training. Sessions concluded with the 30-shot protocol, an assessment of RPE, and a second SSAT conducted 3 min post-intervention (Figure 1). Prior to each DT session, participants received verbal instructions from the lead investigator specifying the movement variation to be applied. These variations were grounded in the theoretical principles of the DL model for motor skill acquisition (Schöllhorn et al., 2012; Oftadeh et al., 2021). The TT protocol was structurally matched to the DT condition in terms of volume, shot distribution, and spatial configuration but was performed without imposed variability. All shots in the TT condition were executed with a standard size 6 basketball, under consistent and repetition-based constraints. Outside the intervention, both groups maintained their regular basketball training schedules. Each session lasted approximately 90 min and included 30 min of technical skill instruction followed by 30 min of continuous full-court (5v5) gameplay. These sessions were conducted independently of the experimental intervention. On non-intervention days, both groups participated in one 90-min basketball training session per week. Each session consisted of 15-min warm-up, 20 min of small-sided games (1v1, 2v2, and 3v3), 25 min of technical skill instruction, and 30 min of full-court (5v5) gameplay. The training structure and workload were standardized and consistently implemented across both groups. Two weeks after the post-test, all subjects participated in the retention tests.

**Table 1:** Shooting variations in DT and TT protocols.

Shooting based variables	Sessions	Traditional shooting training	Differential shooting training
Duration	All	8 weeks	8 weeks
		2 sessions per week	2 sessions per week
		5 × 6 shots five angles and three distances.	5 × 6 shots five angles and three distances.
		~25 min per session	~25 min per session
A. Shooting ball	Session 1	Regular basketball size 6	Shooting with a mini handball
	Session 2	Regular basketball size 6	Shooting with a street football
	Session 3	Regular basketball size 6	Shooting with a size 7 basketball
	Session 4	Regular basketball size 6	Randomized set of 10 shots using all balls from Sessions 1–3
B. Body conditions	Session 5	None	Shooting with the dominant hand while the non-dominant hand is held behind the back
	Session 6	None	One-legged shooting
	Session 7	None	Shooting with the non-dominant hand while the dominant hand rests on the chest
	Session 8	None	Randomized set of 10 shots applying all body constraints from Sessions 1–3
C. Target obstacles	Session 9	None	Shooting from a 20 cm elevated platform
	Session 10	None	Shooting 1 m in front of a hands-up defensive mannequin (D-Man)
	Session 11	None	Shooting at a lowered basket (2.80 m)
	Session 12	None	Randomized set of 10 shots using all target constraints from Sessions 1–3
D. Perceptual conditions	Session 13	None	Shooting with one eye closed
	Session 14	None	Shooting while wearing plastic gloves
	Session 15	None	Shooting while wearing dribbling goggles.
	Session 16	None	Randomized set of 10 shots using all perceptual constraints from Sessions 1–3

#### 2.2.2 Data collection

##### 2.2.2.1 Stationary Shooting Accuracy Test (SSAT)

To evaluate the acute effects of DT on shooting performance, a modified version of the SSAT was employed, based on the protocol established by Pojskic et al. (2018). After completing the standardized warm-up, each participant executed two jump shots from six predetermined court locations, resulting in a total of 12 attempts (Figure 1). The shooting sequence began at the right wing, designated as Position 1. The six positions were spaced across three diagonal distances from the basket: 3.90 m (Positions 1 and 4), 5.30 m (Positions 2 and 5), and 6.75 m (Positions 3 and 6). Participants were allowed unlimited time to complete their attempts; however, standardized verbal cues were used to encourage prompt transitions between positions. Two additional players assisted during the test by retrieving rebounds and returning the ball to the shooter. Shooting performance was assessed by recording the total number of successful shots made during the trial. SSAT tallies (made/missed) were recorded on court using standardized forms; assessors were not blinded to allocation, which we mitigate by using an objective binary outcome and fixed test order.

##### 2.2.2.2 30-shot task and performance measure

In separate sessions, the DTG completed a 30-shot task under four predefined boundary conditions: ball type variations (A), body movement restrictions (B), target modifications (C), and perceptual conditions (D) (Table 1). In contrast, the TTG followed the same 30-shot protocol across all sessions without the incorporation of external constraints. Each successful shot was awarded one point and recorded by the researcher. Total performance scores were computed by summing the number of successful attempts. No time limit was imposed for task completion. During each session, two additional players assisted by retrieving rebounds and returning the ball to the shooter. Once one participant completed the 30-shot task, the next participants began their trial.

##### 2.2.2.3 Rate of perceived exertion (RPE)

To quantify exercise intensity during each training intervention, researchers employed the Borg 10-point RPE scale (Borg, 1998). This validated psychophysiological instrument (Rodríguez-Marroyo and Antoñan, 2015), ranging from 0 (no exertion) to 10 (maximal exertion), allows athletes to self-assess their perceived effort in real time. RPE scores were documented immediately after each conclusion of every training intervention and SSAT.

##### 2.2.2.4 Basketball Jump Shooting Accuracy Test (BJSAT)

**Figure 2:**
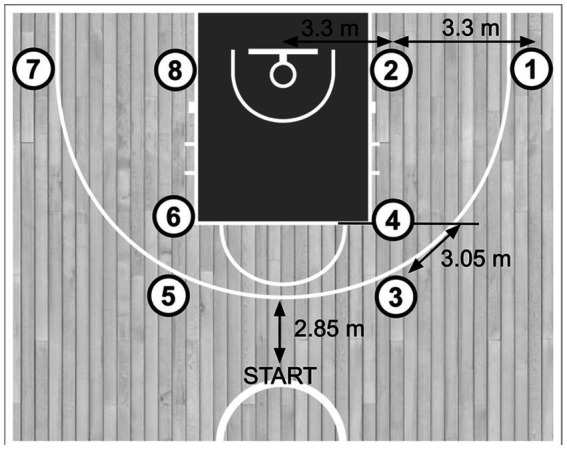
Court layout for the Basketball Jump Shooting Accuracy Test (BJSAT).

To evaluate the effectiveness of the DT program, the Basketball Jump Shooting Accuracy Test (BJSAT) was employed. This protocol was adapted from the modified version adapted from Boddington et al. (2019). Prior to testing, all athletes received a standardized demonstration of the BJSAT, followed by a two-minute warm-up that included shots from the designated locations. The BJSAT consisted of eight predefined shooting positions: four for two-point attempts and four for three-point attempts. Athletes completed an equal number of shots from both the left and right sides of the court to ensure spatial symmetry. Each participant performed two continuous BJSAT trials. Verbal instructions were provided to ensure adherence to the prescribed shooting sequence. Each set comprised 16 jump shot attempts, one from each location, executed in a fixed alternating order between two- and three-point distances to avoid consecutive shots from the same range. Trials began at a designated midpoint between the half-court line and the three-point arc (Figure 2). Two supporting players assisted during testing by retrieving rebounds and returning the ball to the shooter. At each shooting station, athletes were required to place both feet within a 60 cm × 60 cm marked boundary. If a shot was taken with one or both feet outside the area, shot was considered as invalid, the trial continued, but verbal feedback was provided immediately to correct foot placement for subsequent attempts. Athletes were instructed to complete each trial at a maximal pace to replicate game-like tempo. Each set comprised 16 jump-shot attempts, one from each location, executed in a fixed alternating order between two- and three-point distances. Players performed the sequence at a brisk, continuous cadence, guided by standardized verbal prompts to transition promptly between stations; no formal time limit was imposed or recorded. Each shot was rated 0–3 (Table 2). At each test occasion players completed two BJSAT trials. Each trial comprised 16 rated attempts—one shot from each of the 8 locations (4 two-point; 4 three-point) in an alternating sequence. The “2-pts score” equals the sum of eight 0–3 ratings from two-point attempts (4 locations × 2 trials = 8 shots; range 0–24). The “3-pts score” equals the sum of eight 0–3 ratings from three-point attempts (range 0–24). For descriptive purposes we also report the overall total across the 16 shots (range 0–48). Foot-placement faults (feet outside the 60 × 60 cm area) were coded as 0 and not repeated, maintaining a constant number of rated attempts. For analysis, we computed bounded composite scores: a 2-pt total and a 3-pt total (each 0–24; eight rated shots per range), and an overall descriptive total (0–48) across 16 shots. BJSAT ratings (0–3; Table 2) were performed on court according to deterministic criteria; assessors were not blinded to group or time. To limit detection bias, foot-faults were rule-based (coded 0) and the number of rated attempts was constant.

**Table 2:** Scoring criteria for the Basketball Jump Shooting Accuracy Test (BJSAT) (Boddington et al., 2019).

Score	Description
3	The basketball travels through the basket without contacting the rim or backboard.
2	The basketball contacts the rim or backboard before traveling through the basket.
1	The basketball contacts the rim or backboard but does not travel through the basket.
0	The basketball does not contact the rim or backboard and does not travel through the basket.

##### 2.2.2.5 Small-sided game (1v1) shooting performance

To assess the impact of the DT program on individual scoring performance, a SSG format was implemented using 1v1 basketball. This design was selected to replicate game-specific demands while isolating offensive and defensive actions within a controlled setting. Scoring performance was evaluated at three time points: pre-test, post-test, and retention test. Player pairings were determined according to playing position and skill level to ensure competitive parity. These pairings remained constant across all sessions to enable reliable performance comparisons. Although the primary metric was the total number of points scored in 5 min, two trained evaluators independently coded the video to (i) confirm each valid field goal (ball completely passing through the basket from a live, in-bounds possession), (ii) identify rule violations that nullified baskets (traveling, double dribble/carry, out-of-bounds, offensive charge/push), and (iii) verify protocol fidelity (role alternation after each possession; continuous play; exclusion of free throws). Disagreements were resolved by consensus on second viewing. We computed inter-rater reliability for bout-level total points using the intraclass correlation coefficient (ICC), which was 0.92, indicating excellent agreement. Each 1v1 bout was conducted on a half-court following standard basketball regulations, with minor modifications to promote continuous play. Players alternated between offensive and defensive roles after each possession. Free throws were excluded, and possession shifted immediately following a made basket or defensive stop. Each bout lasted 5 min, during which participants were instructed to compete at maximal efforts, according to the previously used basketball SSG format conditions (Clemente et al., 2021). Coaches provided real-time verbal prompts to reinforce effort and support decision-making aligned with game dynamics. Participants were encouraged to employ a diverse set of offensive maneuvers, such as jab steps, shot fakes, and dribble penetrations, to simulate realistic in-game scenarios. Scoring followed a one-point-per-basket system. The total number of points scored during each five-minute bout was recorded as the primary metric of individual scoring performance. All 1v1 bouts were video recorded using digital camera (GoPro Hero9 Black). The camera were positioned 2.5 m above the court and 5.5 m away from the courts to provide an optimal and unobstructed view for analysis. Raters were not formally blinded to group or time during video coding; adjudication used objective criteria (valid basket/violation/protocol fidelity), and bout-level points showed excellent inter-rater agreement (ICC = 0.92).

### 2.3 Statistical analysis

Descriptive statistics (mean ± SD) were computed for all variables. For the longitudinal outcomes—BJSAT two-point score, BJSAT three-point score, and 1 × 1 SSG scoring—we fitted linear mixed models (LMMs) with Group (DTG, TTG), Time (pre, post, retention; categorical), and the Group × Time interaction as fixed effects, and Participant as a random intercept to account for within-subject dependence. The Gaussian family with identity link was used. Models were estimated by restricted maximum likelihood (REML). Planned contrasts were specified on the Time factor (and its interaction) to test pre → post, pre → retention, and post → retention comparisons; in the tables these are denoted a, b, and c, respectively (inter-group estimates in Table 3; intra-group estimates in Table 4). When reporting between-group differences at a given time point, we used the estimated marginal means from the fitted Group × Time model. 95% confidence intervals and *p*-values are reported for all tests. For RPE, an LMM was fitted with Group (DTG, TTG), Session (1–16; categorical), and Condition (A–D; categorical) as fixed effects and Participant as a random intercept; Group × Session and Group × Condition interactions were inspected and retained only if significant. For SSAT (before/after within session) and the 30-shot task, separate LMMs compared groups with Participant as a random intercept and included Session as a fixed effect when appropriate. For each DTG–TTG comparison we computed Cohen’s d by standardizing the model-estimated contrast with the residual SD (*σ*) from the fitted mixed model; 95% CIs for *d* were obtained by standardizing the contrast CI limits. All mixed models were fitted by REML using all available observations under a Missing at Random assumption. Model assumptions were checked via residual-versus-fitted, Q–Q, and scale–location plots, and by influence diagnostics. Where near-singular fits occurred, we repeated the analysis with a random-intercept–only specification; Statistical significance was set at *α* = 0.05. Analyses were conducted in Jamovi (v1.2.27).

**Table 3:** Fixed effects and estimated marginal means for inter-group comparisons across testing moments.

Var	Comp	DTG		TTG		AIC	R-squared conditional	95% CI	SE	*p*-value	Cohen’s *d*	95% CI
		EMM	SE	EMM	SE							
2-pts score	a	12.6	0.71	12.3	0.78	495.567	0.599	3.49 (2.32, 4.66)	0.599	<0.001	0.13	(−0.53, 0.80)
	b	17.3	0.71	14.6	0.78	495.567	0.601	1.99 (0.82, 3.17)	0.601	0.001	1.13	(0.48, 1.93)
	c	14.9	0.76	14.0	0.77	495.567	0.593	−1.5 (−3.00, −0.82)	0.593	0.044	0.38	(−0.27, 1.08)
3-pts score	a	10.5	0.70	10.1	0.79	507.161	0.738	2.37 (0.93, 3.81)	0.738	0.006	0.13	(−0.53, 0.81)
	b	13.1	0.70	12.3	0.79	507.161	0.74	1.92 (0.48, 3.369)	0.74	0.035	0.26	(−0.39, 0.95)
	c	12.9	0.77	11.6	0.77	507.161	0.744	0.45 (−1.96, 1.40)	0.744	1.000	0.43	(−0.22, 1.13)
1 × 1 SSG score	a	5.99	0.75	5.27	0.85	520.433	0.784	4.77 (3.24, 6.30)	0.784	<0.001	0.22	(−0.43, 0.91)
	b	11.65	0.75	9.14	0.85	520.433	0.786	3.36 (1.83, 4.90)	0.786	<0.001	0.78	(0.14, 1.53)
	c	9.20	0.79	9.14	0.82	520.433	0.79	−1.4 (−3.91, 0.75)	0.790	0.241	0.13	(−0.53, 0.80)

Var = Variable; Comp, Comparison; DTG, Differential Training Group; TTG, Traditional Training Group; EMM, Estimated Marginal Means; SE, Standard Error; AIC, Akaike Information Criterion; CI, Confidence intervals; 2-pts, Two-point; 3-pts, Three-point; 1×1 SSG, Small Sided Game; a, Pre-test vs. Post-test; b, Pre-test vs. Retention test; c, Post-test vs. Retention test; 2-pt: *σ* = 2.33; 3-pt: *σ* = 2.957; 1 × 1 SSG: *σ* = 3.138.

**Table 4:** Fixed effects and descriptive statistics for intra-group comparisons across testing sessions.

Variable	Group	Comp	Mean Diff (*β*)	SE	95% CI	*p*-value
2-pts score	DTG	a	1.2	0.357	(0.5, 1.9)	0.002
	DTG	b	1.0	0.357	(0.3, 1.9)	0.005
	DTG	c	−0.2	0.357	(−0.9, 0.5)	0.56
2-pts score	TTG	a	0.4	0.357	(−0.3, 1.1)	0.23
	TTG	b	0.2	0.357	(−0.5, 0.9)	0.37
	TTG	c	−0.2	0.306	(−0.8, 0.4)	0.64
3-pts score	DTG	a	0.9	0.306	(0.3, 1.5)	0.01
	DTG	b	0.7	0.306	(0.1, 1.3)	0.03
	DTG	c	−0.2	0.306	(−0.8, 0.4)	0.45
3-pts score	TTG	a	0.1	0.306	(−0.5, 0.7)	0.74
	TTG	b	−0.1	0.306	(−0.7, 0.5)	0.83
	TTG	c	−0.2	0.306	(−0.8, 0.4)	0.59
1×1 SSG score	DTG	a	2.3	0.663	(1.8, 3.6)	0.001
	DTG	b	2.1	0.663	(0.8, 3.4)	0.002
	DTG	c	−0.2	0.663	(−1.5, 1.1)	0.71
1×1 SSG score	TTG	a	0.5	0.663	(−0.8, 1.8)	0.42
	TTG	b	0.3	0.663	(−1.0, 1.6)	0.6
	TTG	c	−0.2	0.663	(−1.5, 1.1)	0.73

DTG, Differential Training Group; TTG, Traditional Training Group; SE, Standard Error; 2-pts, Two-point; 3-pts, Three-point; 1×1 SSG, Small Sided Game; a, Pre-test vs. Post-test; b, Pre-test vs. Retention test; c, Post-test vs. Retention test.

## 3 Results

Diagnostics were acceptable and complete-case sensitivity analyses did not change the inferences. The linear mixed model revealed a significant time effect on 2-point shooting accuracy (*p* < 0.001), with the DTG presenting significantly greater improvements at post-test compared to the TTG (*β* = −2.48, *p* = 0.042). These improvements persisted during the retention phase (*p* = 0.001), although a slight decrease was observed between post-test and retention (*p* = 0.044). Regarding 3-point shooting, both DTG and TTG groups demonstrated performance enhancement over time (*p* = 0.004), but no statistically meaningful differences emerged between groups. Scores from 1 × 1 SSG increased over time for the sample (time effect: *p* < 0.001). Between-group differences were not significant at retention; DTG showed numerically larger gains (DTG: pre → post *β* = 2.3, *p* = 0.001; pre → retention *β* = 2.1, *p* = 0.002) compared with TTG (pre → post *β* = 0.5, *p* = 0.42; pre → retention *β* = 0.3, *p* = 0.60). In the 30-shot assessment, task conditions significantly affected outcomes, with the “Shooting ball” variation (Condition A) yielding superior results. Conversely, accuracy significantly decreased under the “Body” (B), “Target obstacle” (C), and “Perceptual” (D) constraints, with notable differences such as between D and A (*β* = −11.76, *p* = 0.020). Additionally, session number exerted a consistent and significant influence (*p* < 0.001). No relevant differences were observed in stationary shooting accuracy before the 30-shot test, suggesting baseline equivalence across conditions. After completing the 30-shot task, only a borderline significant improvement was found in session 5 (*p* = 0.051), indicating a possible short-term adaptation effect. Rate of perceived exertion (RPE) varied across sessions (time effect: *p* < 0.001). Critically, a group main effect indicated lower RPE in DTG than TTG (1.47 ± 0.18 vs. 2.43 ± 0.18; *β* = −0.96, 95% CI [−1.47, −0.46]; *p* < 0.001; Table 5). No significant group × session or group × condition interactions were detected. Within-group changes across the intervention were −0.8 ± 0.19 for DTG (*p* = 0.040) and −0.4 ± 0.16 for TTG (*p* = 0.110) (Table 6).

**Table 5:** Fixed effects and estimated marginal means for inter-group comparisons.

Var	Comp	DTG	TTG	AIC	R-squared Cond	95% CI	SE	*p*-value	Cohen’s *d*	95% CI
SSAT before the task	DTG/TTG	5.62 ± 0.32	5.07 ± 0.34	1,527.87	0.324	0.44 (0.05, 0.84)	0.195	0.027	0.06	(−0.38, 0.50)
30 shooting task	DTG/TTG	15.2 ± 0.80	12.0 ± 0.84	2,035.06	0.508	3.11 (1.59, 4.63)	0.87	<0.001	1.09	(0.56, 1.61)
SSAT after the task	DTG/TTG	5.98 ± 0.33	5.38 ± 0.34	1,542.27	0.3266	0.31 (−0.05, 0.66)	0.182	0.091	0.18	(−0.27, 0.64)
RPE	DTG/TTG	1.47 ± 0.18	2.43 ± 0.18	938.62	0.473	−0.96 (−1.47, −0.46)	0.254	<0.001	−1.33	(−1.72, −0.94)

Var, Variable; Comp, Comparison; DTG, Differential Training Group; TTG, Traditional Training Group; AIC, Akaike Information Criterion; SE, Standard Error; SSAT, Stationary Shooting Accuracy Test; RPE, Rate of Perceived Exertion; SSAT pre: *σ* = 1.529; SSAT post: *σ* = 1.554; 30-shot: *σ* = 2.87; RPE: *σ* = 0.729.

**Table 6:** Fixed effects and descriptive statistics for intra-group comparisons.

Variable	Group	Mean Diff (*β*)	SE	95% CI	*p*-value
SSAT before the task	DTG	2.1	0.38	(1.1, 3.1)	0.01
SSAT before the task	TTG	1.2	0.35	(0.4, 2.0)	0.04
30 shooting task	DTG	3.2	0.47	(1.5, 4.9)	0.01
30 shooting task	TTG	1.1	0.41	(0.1, 2.1)	0.04
SSAT after the task	DTG	1.8	0.32	(0.8, 2.8)	0.02
SSAT after the task	TTG	1.1	0.30	(0.2, 2.0)	0.04
RPE	DTG	−0.8	0.19	(−1.5, −0.1)	0.04
RPE	TTG	−0.4	0.16	(−1.0, 0.2)	0.11

DTG, Differential Training Group; TTG, Traditional Training Group; SE, Standard Error; SSAT, Stationary Shooting Accuracy Test; RPE, Rate of Perceived Exertion.

## 4 Discussion

The present study aimed to investigate the effects of DT on mid-range and long-range jump shot accuracy, as well as on shooting performance during 1 × 1 SSG, in comparison to TT, which remains the prevailing approach in applied settings.

Results from linear mixed model analyses revealed that DT elicited significant improvements in 2-pts shooting accuracy. These gains remained statistically significant at the retention stage, despite a modest post-intervention decline. For 3-pts shooting, the DTG exhibited improvements during the intervention phase. However, no significant between-group differences were detected at the post-retention assessment, and within-group improvements were not statistically significant at retention. Shooting performance in the 1 × 1 SSG task improved across the sample; however, no between-group difference was detected at retention. The DTG’s gains were numerically larger but did not reach significance, which may reflect the specificity gap between the isolated shooting practice and the integrated perceptual-motor demands of 1 × 1 play. Importantly, DT was associated with lower RPE relative to TT (Table 5), despite matched training volume and shot distribution, suggesting potential cognitive–perceptual and self-regulatory benefits of DT environments.

These findings are consistent with previous research demonstrating the effectiveness of DL in enhancing skill acquisition. Multiple studies have shown that DL yields superior motor learning outcomes compared to traditional, repetitive training approaches (Tassignon et al., 2021). This aligns with the work of Schöllhorn et al. (2012) and Schöllhorn et al. (2022) who emphasize that introducing random variations (stochastic perturbations) into practice promotes greater movement variability and adaptability, thereby facilitating more robust skill development and accelerating learning. For example, Oftadeh et al. (2021) reported that futsal players who underwent a three-month DL based intervention with external focus cues demonstrated superior skill retention and transfer compared to those receiving conventional training, suggesting that the effects of DL interventions may strengthen over time. Extending this evidence to other sports, Wagner and Müller (2008) found that DL improved the qualitative execution of handball throwing. Their findings suggest that DL not only enhances motor performance but also promotes a closer alignment between movement patterns and intended outcomes. These insights underline the value of integrating DL principles into contemporary training programs to foster adaptable and transferable motor skills.

The DT protocol, which specifically targeted improvements in basketball shooting accuracy showed a greater effect observed for 2-pts shooting scores (*p* < 0.001), compared to 3-pts shooting scores (*p* < 0.006), likely reflects the well-documented distance–accuracy trade-off in basketball. Previous research has consistently shown that 2-pts shots are more accurate than 3-pts attempts (Kilinç, 2008; Özmen, 2016), mirroring in-game patterns where closer-range shots yield higher success rates. Longer-distance shots require an increased release height, along with corresponding adjustments in velocity and angle, to maintain shot stability and accuracy (Okazaki and Rodacki, 2012; Cabarkapa et al., 2023). These biomechanical adjustments, although required for long-range shooting, can negatively affect performance consistency (Kilinç, 2008). Our findings align with this interpretation. Two- and three-point shooting scores in the DTG evolved at different rates over the 8-week DT period (Table 2), indicating distinct yet complementary adaptations to the specific demands of the training stimulus.

By engaging participants in DT tasks that challenged their adaptability, DL approach enhances not only the shooting skills but also the ability to retain 2-pts shooting over time. Numerous studies have examined the effects of DL on motor skill acquisition and, importantly, on retention—the ability to maintain improved performance after a period without practice (Henz and Schöllhorn, 2016). For instance, soccer players maintained or even improved their performance up to 4 weeks post-intervention, while the repetitive group’s performance dropped back to baseline within 2 weeks after training ended (Schollhorn et al., 2009). At retention, especially under pressure, the basketball free-throw intervention group significantly outperformed the repetitive training group, indicating superior retention and transfer of skill under realistic conditions (Lattwein et al., 2014). Similar retention benefits for DL over repetitive training have been observed in handball (Wagner and Müller, 2008), volleyball (Römer et al., 2009), track and field (Henz and Schöllhorn, 2016), ice-skating (Savelsbergh et al., 2010), and hockey (Beckmann and Schöllhorn, 2006). In these studies, DL groups showed continued improvement or maintained gains at retention tests, while repetitive groups often regressed.

The observed improvements in 1 × 1 SSG shooting performance from pre- to post-intervention likely reflect enhanced real-time decision-making under conditions of game-like variability. Effective shot creation in such dynamic contexts requires players to continuously perceive and interpret environmental cues and to make rapid, context-specific decisions.

Although DL has been shown to enhance skill retention and transfer in team sports (Santos et al., 2016), our findings indicate that DT did not significantly improve 1 × 1 SSG scoring performance during the retention phase. Warren’s (2006) behavioral dynamics framework suggests that adaptive movement patterns emerge through ongoing interaction with environmental conditions, reinforcing the notion that athletes detect opportunities to act in real time. Nevertheless, it is important to acknowledge that the 1 × 1 SSG task, while valuable for assessing shot accuracy, differs substantially in both physical intensity, emotional challenges, and tactical complexity from the 5v5 competitive basketball contexts in which our participants typically need to train. Further research is warranted to determine how DT interventions translate to performance outcomes in full-game settings. It is also plausible that the limited retention-phase improvement observed in the 1 × 1 SSG task reflects a specificity mismatch between the training intervention—focused on isolated shooting conditions—and the assessment task, which demanded integrated perceptual-motor responses under dynamic, opponent-based conditions. As such, future implementations of DT might benefit from considering not only the amount of noise but also the structure and the area of noise, e.g., in terms of similarity of exercise and target movement, including fluctuating game-based scenarios, to better cover the possible space of solutions to foster interpolation instead of extrapolation (Schöllhorn, 2000).

Our findings further underscore the role of movement and exercise “noise” as a key factor in skill acquisition. This was evident in the acute responses observed during the intervention phase (Tables 3, 4). Within the context of DT, variability in shooting execution, plays a dual role: fostering rapid adaptation and supporting reinforcement learning processes. Chen et al. (2017) identified motor noise as a fundamental component of motor learning, particularly through its interaction with decision-making during exploratory learning. This capacity to adapt under noisy conditions constitutes a strategic advantage in motor learning (van Beers, 2009). Our 30-shot task results (Table 3) reinforce this perspective. Shooting scores were significantly higher in the DTG than in the TTG group (15.2 ± 0.80 vs. 12.0 ± 0.84, *p* < 0.001). Moreover, prior research has shown that individual differences in motor noise correlate with adaptation rates (van der Vliet et al., 2018). This finding is consistent with our intragroup results (Table 4), where the DT exhibited a significantly greater performance increase compared to the TT. These results suggest that adaptive responses are modulated by both planning and execution variability, underscoring the need for an integrated motor learning framework that accounts for multiple sources of noise.

Interestingly, the acute effects of DT were not immediately distinguishable. Both the DTG and TTG groups demonstrated significant gains in shooting accuracy (*β* = 1.8, *p* = 0.02; *β* = 1.1, *p* = 0.04, respectively). This observation aligns with findings from systematic reviews, which indicate that youth basketball shooting performance is shaped by multiple factors, including distance, fatigue, defensive pressure, visual and acoustic information, etc. (França et al., 2021). These sources of variability underscore the importance of designing training interventions that simulate competitive conditions to promote transferability.

Regarding perceived effort, RPE was significantly lower in the DTG group compared to the TTG group after training (*p* < 0.001), despite comparable improvements in performance. This finding supports prior evidence that training environments and psychological factors play a critical role in shaping perceived exertion (Smits et al., 2014). For instance, motivational stimuli such as music can reduce RPE and enhance performance sustainability (Clark et al., 2021). Our findings further corroborate the principles of the DL framework, which emphasizes self-regulation and adaptability. Jarraya et al. (2012) and Vandoni et al. (2017) suggest that RPE encompasses not only physical fatigue but also cognitive and emotional factors that influence learning. Pageaux (2016) further highlights that teaching strategies addressing mental fatigue and emotional load can optimize motor learning outcomes. Accordingly, RPE should be regarded not solely as a measure of physical effort, but as a multidimensional tool for informing the design of effective training protocols.

Future research on DT in sports should further investigate the offensive and defensive performance of basketball players across distinct competitive formats, such as 3 × 3 and 5v5 games. Moreover, the interplay among athlete enjoyment, engagement, and RPE within differential learning contexts warrants systematic examination. Comparative investigations contrasting the efficacy of traditional and nonlinear pedagogical frameworks across diverse sporting environments would provide deeper insights into the mechanisms and practical implications of DL. The near-significant *p*-value for 3-pts score could warrant further investigation with larger samples or more statistical power.

## 5 Conclusion

This study contributes to the growing body of evidence supporting DL as an effective approach for enhancing basketball skill acquisition, particularly when operationalized as DT. This study supports DL (operationalized as DT) as an effective approach for enhancing two-point shooting accuracy. In 1 × 1 SSG scoring, both groups improved over time, but no between-group difference was evident at retention. DT was consistently associated with lower perceived exertion than TT. Furthermore, DT is associated with lower RPE, indicating benefits that extend across both physical and cognitive domains of performance. From a motor learning perspective, these findings align with theoretical models that emphasize the role of movement variability, motor noise, and perception–action coupling in facilitating adaptive skill development. The distinct rates of improvement observed in mid- and long-range shooting suggest that athletes adapt in skill-specific ways to the variable demands imposed by DT. For sport scientists, these results position DT as a research-informed and ecologically valid training method capable of enhancing functional performance while mitigating both mental and physical load. For sports practitioners, the transfer of skills acquired within a DT environment to real-world performance contexts is influenced by the selection and regulation of motor noise levels. The individual capabilities of each athlete may constitute a critical constraint, as the intensity and informational demands of real match play can evoke distinct cognitive, physical, and motor adaptations that shape performance outcomes. Nevertheless, the limited transfer effects observed in post-retention SSG outcomes highlight the need for further investigation into how DT interventions translate to more complex and dynamic team play contexts.

## Data Availability

The raw data supporting the conclusions of this article will be made available by the authors without undue reservation.
